# Successful pulsed field ablation for refractory premature ventricular contractions using a novel dual-energy focal catheter: a case report

**DOI:** 10.1093/ehjcr/ytag689

**Published:** 2026-09-10

**Authors:** Kohei Ukita, Roman Mamaev, Sorin Stefan Popescu, Roland Richard Tilz

**Affiliations:** Department of Rhythmology, University Heart Center Lübeck, University Hospital Schleswig-Holstein, Ratzeburger Allee 160, 23562 Lübeck, Germany; Department of Rhythmology, University Heart Center Lübeck, University Hospital Schleswig-Holstein, Ratzeburger Allee 160, 23562 Lübeck, Germany; Department of Rhythmology, University Heart Center Lübeck, University Hospital Schleswig-Holstein, Ratzeburger Allee 160, 23562 Lübeck, Germany; German Center for Cardiovascular Research (DZHK), Partner Site Lübeck, Germany; Department of Rhythmology, University Heart Center Lübeck, University Hospital Schleswig-Holstein, Ratzeburger Allee 160, 23562 Lübeck, Germany; German Center for Cardiovascular Research (DZHK), Partner Site Lübeck, Germany

**Keywords:** Case report, Left ventricular outflow tract, Premature ventricular contraction, Pulsed field ablation, Radiofrequency ablation, Right ventricular outflow tract

## Abstract

**Background:**

Radiofrequency ablation (RFA) is an established therapy for symptomatic premature ventricular contractions (PVCs), but arrhythmias from deep intramural substrates may remain refractory. Pulsed field ablation (PFA) is a nonthermal modality with myocardial selectivity, and dual-energy focal catheters allow delivery of both RFA and PFA using a single device. We report successful PFA for RFA-refractory PVCs using this technology.

**Case Summary:**

An 81-year-old man with ischaemic cardiomyopathy and symptomatic monomorphic PVCs was referred after failed prior RFA of both right ventricular outflow tract (RVOT) and left ventricular outflow tract (LVOT). During repeat ablation, activation mapping identified earliest activation at the septal RVOT and anterior LVOT. Using a dual-energy focal catheter, sequential RFA and PFA were delivered at both sites. In the RVOT, PFA caused only transient suppression with recurrence of PVCs. In the LVOT, the first PFA application failed to eliminate PVCs, whereas additional applications resulted in immediate and sustained suppression. No complications occurred, and the patient remained free of PVCs during follow-up.

**Discussion:**

This case demonstrates the feasibility of dual-energy focal catheter–based PFA for ventricular arrhythmias refractory to RFA. The elimination of PVCs after repeated PFA suggests that multiple PFA applications may enhance lesion formation in deep intramural substrates. However, this observation should be interpreted cautiously because histological confirmation was unavailable and alternative explanations, including cumulative energy exposure and autonomic variability during conscious sedation, cannot be excluded. Further studies are required to define optimal energy strategy, lesion characteristics, and long-term outcomes of dual-energy ablation for ventricular arrhythmias.

Learning points

- Pulsed field ablation (PFA) using a novel dual-energy focal catheter may be a feasible therapeutic option for refractory premature ventricular contractions.
- In cases of presumed deep intramural ventricular arrhythmias, repeated PFA applications may enhance lesion formation and arrhythmia suppression.
- Dual-energy ablation catheters enable precise colocalized delivery of radiofrequency energy and PFA without catheter exchange.

## Introduction

Radiofrequency ablation (RFA) is a reasonable treatment option for frequent, symptomatic, drug-refractory monomorphic premature ventricular contractions (PVCs).^1^ However, for PVCs refractory to conventional RFA, advanced techniques may be required.^2^

Pulsed field ablation (PFA), a nonthermal ablation modality based on irreversible electroporation, has emerged as a promising alternative energy source with myocardial selectivity and a favourable safety profile.^3,4^ Recently, a novel dual-energy focal ablation catheter, capable of delivering both RFA and PFA using a single device, has been introduced into clinical practice.^5–8^

In this report, we describe a case of refractory PVCs successfully treated with PFA using a novel dual-energy focal catheter.

## Summary figure

**Figure:**
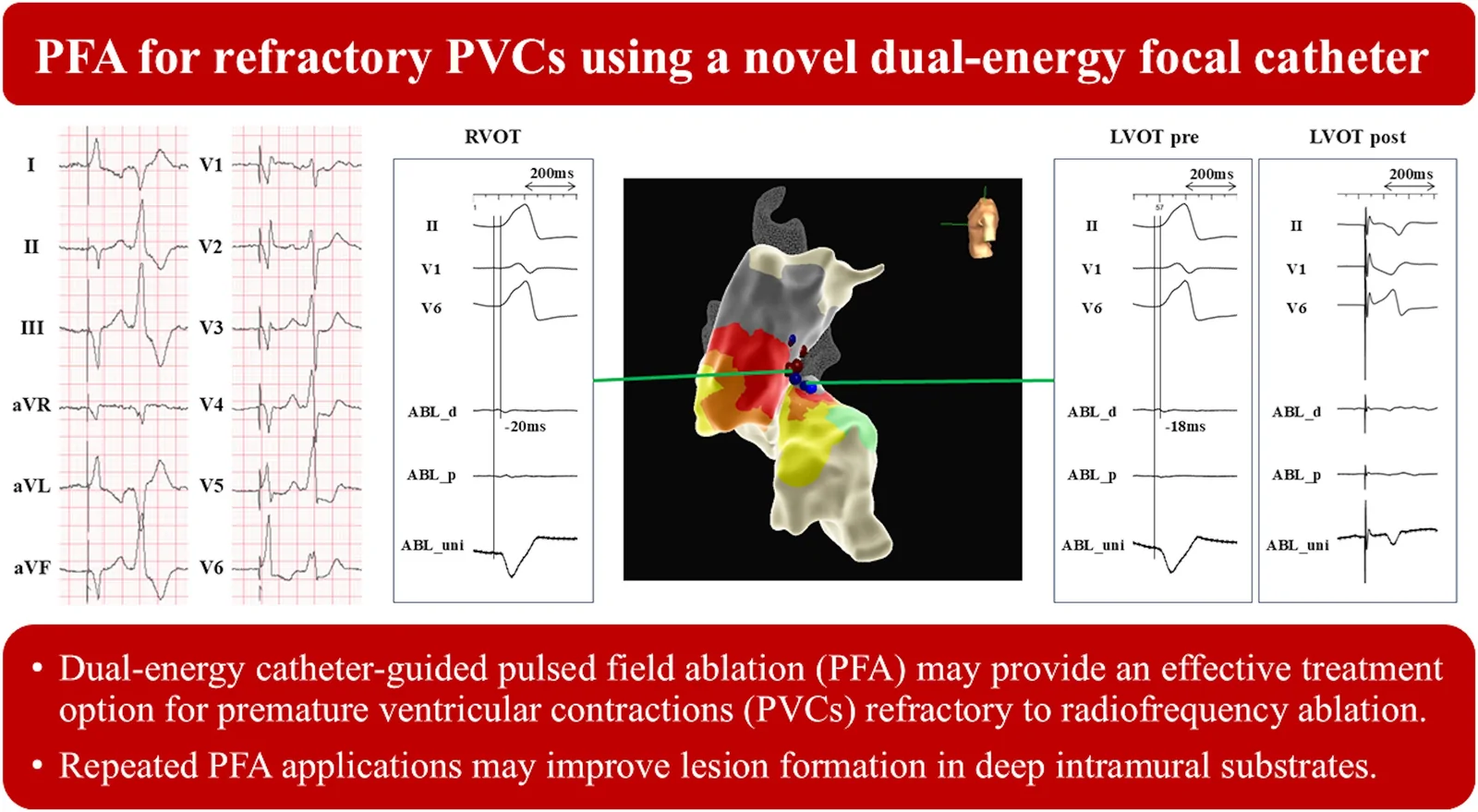
PFA for refractory PVCs using a novel dual-energy focal catheter.

## Case report

**Figure 1:**
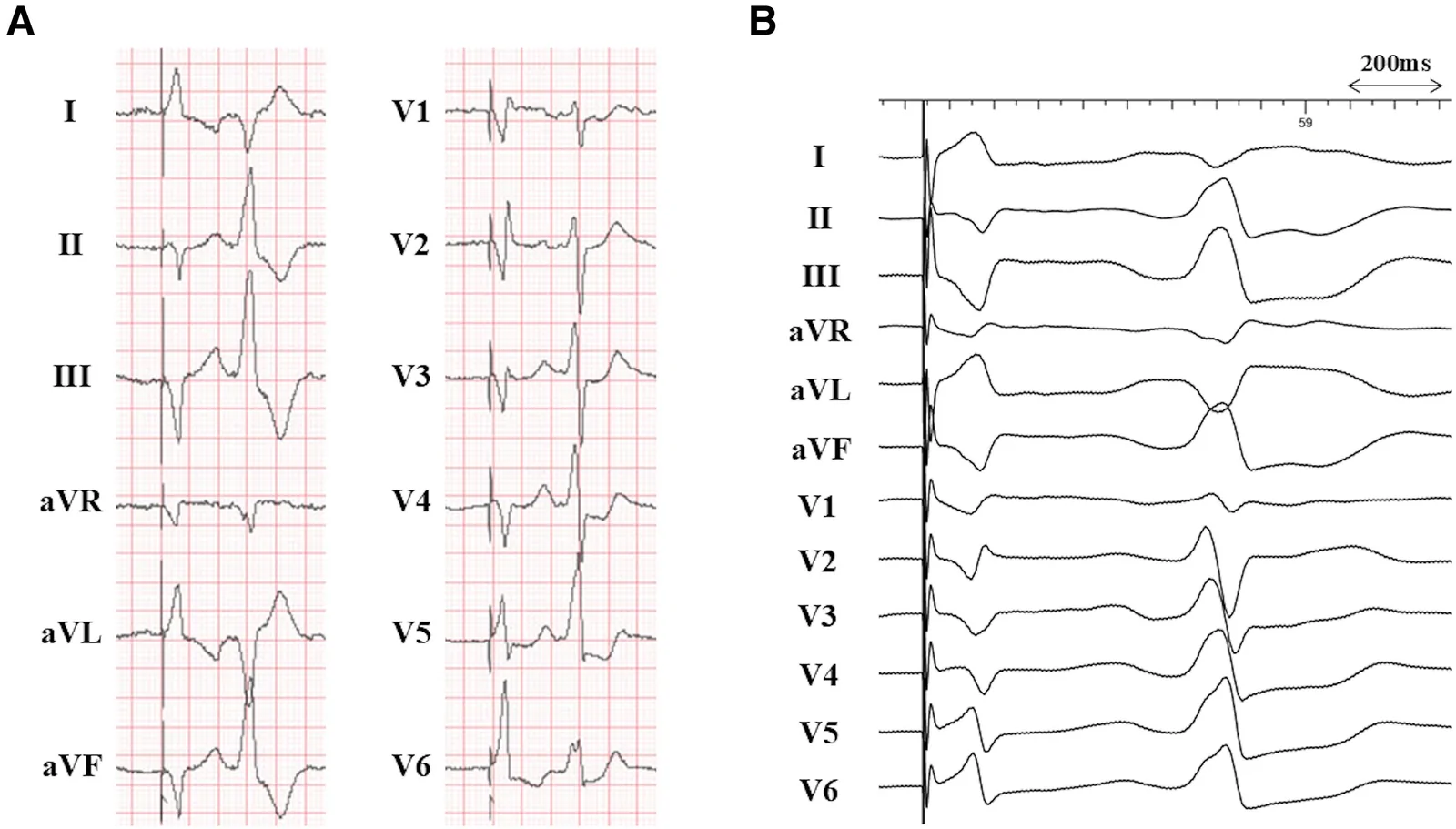
Twelve-lead electrocardiograms before and during the repeat ablation procedure. (*A*) Twelve-lead electrocardiogram before the repeat ablation procedure showing premature ventricular contractions (PVCs) with an inferior axis and a transition zone between leads V3 and V4 (25 mm/s, 10 mm/mV). (*B*) Twelve-lead electrocardiogram at the beginning of the repeat ablation procedure demonstrating PVCs with an inferior axis and a transition zone between leads V2 and V3.

An 81-year-old man with ischaemic cardiomyopathy (left ventricular ejection fraction: 35%) and permanent atrial fibrillation was referred to our hospital for management of symptomatic recurrent PVCs. The patient had undergone dual-chamber pacemaker implantation with left bundle branch pacing, transcatheter bicaval valve (TricValve^TM^, P&F Products & Features) implantation for severe tricuspid regurgitation, and subsequently underwent RFA of both right ventricular outflow tract (RVOT) and left ventricular outflow tract (LVOT) at another hospital as the initial ablation procedure for symptomatic monomorphic PVCs with a burden of 24% on Holter monitoring. According to the available procedural records, RFA was performed using an irrigated, contact-force sensing catheter (THERMOCOOL SMARTTOUCH^TM^ SF, Biosense Webster) at 40 W during the initial ablation procedure. However, the PVCs were not suppressed by ablation and were resistant to medical therapy, prompting a repeat ablation procedure at our institution. A 12-lead electrocardiogram (ECG) prior to the repeat ablation procedure revealed monomorphic PVCs with an inferior axis and a transition zone between leads V3 and V4 (*Figure 1A*), consistent with the morphology of the PVCs targeted during the initial ablation procedure.

**Figure 2:**
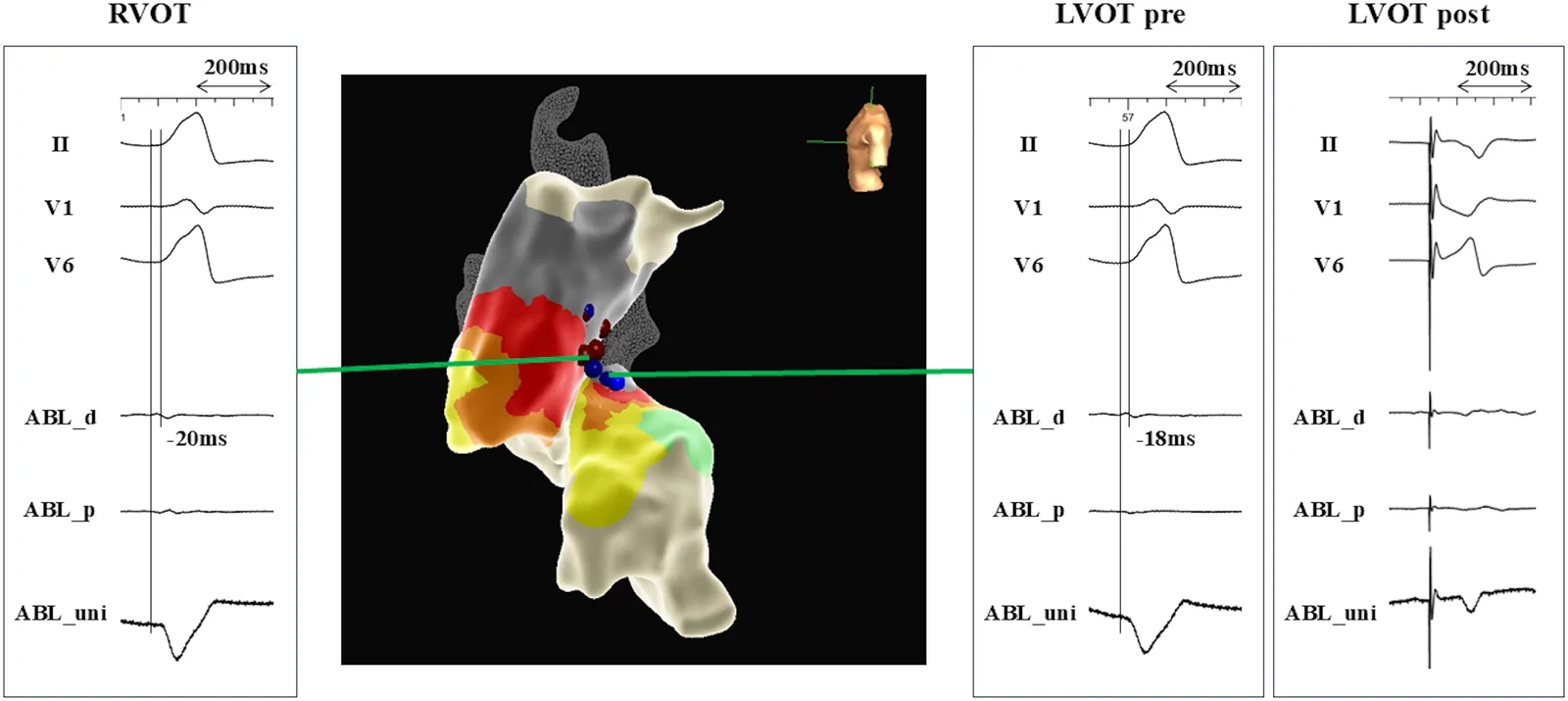
A combined activation map of the right ventricular outflow tract (RVOT) and left ventricular outflow tract (LVOT) during spontaneous PVCs obtained with HD Grid (left lateral and cranial view). Within the RVOT, the earliest ventricular activation was at the septal aspect, preceding the QRS onset by approximately 20 ms. Initial radiofrequency applications were delivered at the site of earliest activation followed by pulsed field ablation (PFA). Within the LVOT, the earliest ventricular activation was at the anterior aspect, preceding the QRS onset by approximately 18 ms. Radiofrequency applications were delivered at the site of earliest activation followed by PFA. Intracardiac electrograms recorded after PFA delivery at the successful ablation site in the LVOT are also shown. LVOT, left ventricular outflow tract; RVOT, right ventricular outflow tract.

Five months after the initial ablation procedure, repeat ablation was performed under conscious sedation using a three-dimensional (3D) mapping system (EnSite^TM^ X, Abbott) (*Table 1*). At baseline, frequent monomorphic PVCs were observed. These PVCs had an inferior axis, and the transition zone was between V2 and V3 (*Figure 1B*), which was slightly earlier than the V3–V4 transition observed on the preprocedural 12-lead ECG; however, they were considered consistent with the same origin, possibly reflecting a slight shift in the exit site. Activation mapping of the RVOT with a high-density mapping catheter (Advisor^TM^ HD Grid, Abbott) during spontaneous PVCs demonstrated the earliest ventricular activation at the septal aspect of the RVOT, preceding the QRS onset by approximately 20 ms (*Figure 2*). Ablation was performed using the dual-energy, focal ablation catheter (TactiFlex^TM^ Duo, Abbott). Initial RFA applications were delivered at the site of earliest activation with adequate contact force (15 g) and power settings (40 W) followed by PFA applications using the manufacturer's standard (‘normal’) biphasic waveform (2400 V). Although transient suppression of PVCs was observed following multiple PFA applications, they recurred shortly thereafter. Subsequently, activation mapping of the LVOT was obtained via transaortic approach and demonstrated the earliest ventricular activation at the anterior aspect of the LVOT, preceding the QRS onset by approximately 18 ms (*Figure 2*). Initial RFA applications were delivered at the site of earliest activation with adequate contact force (15 g) and power settings (40 W), followed by PFA applications with the standard biphasic waveform (2400 V). PVCs persisted after the first PFA application (five bursts at 2400 V); however, they disappeared immediately after the second application (five bursts at 2400 V) at the same site. No further spontaneous or inducible PVCs were observed during a waiting period with isoproterenol infusion and programmed stimulation.

**Table 1:** Timeline of events

5 months before repeat ablation	Initial ablation procedure for frequent symptomatic premature ventricular contractions (PVCs) was performed using radiofrequency energy.
1 month before repeat ablation	The patient was referred to our hospital due to persistent symptomatic PVCs despite prior ablation.
Repeat ablation	Repeat ablation procedure for symptomatic PVCs was performed using a novel dual-energy focal catheter.
The day after repeat ablation	The patient was discharged without complications.
1 month after repeat ablation	No recurrence of PVCs was observed on a 12-lead electrocardiogram, and the patient remained asymptomatic.

Pacemaker interrogation before and after the procedure demonstrated no significant changes in sensing amplitudes, pacing thresholds, and lead impedance without device malfunction. No complications occurred during the procedure. After the procedure, no recurrence of PVCs was observed on monitoring, and the patient was discharged the following day. At the 1-month follow-up, no recurrence of PVCs was observed on a 12-lead electrocardiogram, and the patient remained asymptomatic (*Table 1*).

The patient provided written informed consent for the ablation procedure and agreed to the publication of his case details and images in this report.

## Discussion

This case demonstrates the feasibility of dual-energy focal catheter–based PFA for refractory PVCs. Although several reports have described dual-energy ablation for refractory ventricular arrhythmias,^5,8^ the present case provides an additional procedural observation by documenting successful elimination of PVCs after repeated PFA applications.

Experimental studies using a dual-energy catheter (Dual Energy THERMOCOOL SMARTTOUCH^TM^ SF, Biosense Webster) have shown that sequential application of RFA and PFA resulted in deeper lesion formation, possibly because RFA-induced reductions in tissue impedance and oedema enhance electrical conductivity and facilitate deeper PFA penetration.^9^ In the present case, on the other hand, PFA delivered following RFA at the LVOT did not suppress the PVCs; however, additional PFA applications resulted in their elimination. This finding raises the possibility that, with the TactiFlex Duo, repeated PFA applications may contribute to more effective lesion formation in selected intramural substrates than a single PFA application delivered after RFA. Animal studies using the TactiFlex Duo have also suggested, in a conference abstract, that increasing the number of PFA bursts may create deeper lesions than a single PFA application.^10^ Further investigation is warranted to evaluate the safety, lesion characteristics, and long-term efficacy of multiple PFA bursts using the TactiFlex Duo, as well as their combination with RFA for ventricular arrhythmias.

Our findings should be interpreted cautiously. Without histological confirmation, cardiac magnetic resonance imaging, or direct assessment of lesion depth, the apparent benefit of repeated PFA remains hypothesis-generating. The observed effect may simply reflect cumulative energy delivery from sequential RFA and repeated PFA rather than a specific advantage of repeated PFA itself. Spontaneous variability in PVC frequency or autonomic influences during conscious sedation also cannot be excluded.

An additional question is why PFA alone was not performed. Because our experience with ventricular PFA remains limited, our current practice with the TactiFlex Duo is to combine RFA and PFA, particularly in patients with prior unsuccessful RFA. Accordingly, this procedure was intended to evaluate the complementary use of both energy sources.

This report has several limitations. First, as a single-case report, the findings cannot be generalized, and no definitive conclusions regarding lesion mechanisms or optimal energy strategy can be drawn. Second, the relative contribution of RFA and PFA to the final clinical outcome cannot be determined because both energy sources were intentionally applied in sequence. Third, follow-up rhythm assessment consisted of clinical evaluation and a single 12-lead ECG without repeat Holter monitoring; therefore, asymptomatic or intermittent PVC recurrence cannot be excluded. Fourth, coronary venous mapping, including mapping of the great cardiac vein and anterior interventricular vein, was not performed. Given the patient's advanced age and multiple comorbidities, we adopted a simplified approach targeting the earliest accessible endocardial sites. Nevertheless, such mapping might have provided additional evidence to better define the true site of origin of the PVCs.

## Conclusions

Successful elimination of PVCs occurred after repeated PFA applications following unsuccessful RFA and an initial ineffective PFA application. Although this procedural observation raises the hypothesis that repeated PFA delivery may provide incremental benefit in selected refractory ventricular substrates, this interpretation remains speculative and requires validation in larger clinical and mechanistic studies.

## Lead author biography


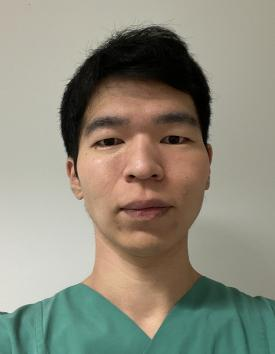


Dr. Kohei Ukita graduated from Osaka University in 2015. After completing his residency, he worked at Osaka Rosai Hospital. Since 2025, he is an Electrophysiology Fellow at the University Hospital Schleswig-Holstein.

## Data Availability

No new data were generated or analysed in support of this study.
